# Liposome-delivered ATP effectively protects the retina against ischemia-reperfusion injury

**Published:** 2010-12-28

**Authors:** Galina Dvoriantchikova, David J. Barakat, Eleut Hernandez, Valery I. Shestopalov, Dmitry Ivanov

**Affiliations:** 1 Bascom Palmer Eye Institute, Department of Ophthalmology, University of Miami Miller School of Medicine, Miami, FL; 2 Department of Molecular, Cell and Developmental Biology, University of Miami Miller School of Medicine, Miami, FL; 3 Vavilov Institute of General Genetics RAS, Moscow, Russian Federation

## Abstract

**Purpose:**

We investigated the effect of ATP (ATP) encapsulated in liposomes (ATP-liposomes) on the level of inflammation and neuronal death in the retina induced by ischemia reperfusion (IR).

**Methods:**

Primary retinal ganglion cells treated with ATP-liposomes, empty liposomes, and phosphate buffer solution (PBS) were deprived of oxygen and glucose (OGD) for 6 h in vitro, in an anaerobic chamber. Plates were assessed for the proportion of necrotic versus apoptotic cells and for cell survival 12 h after OGD. For in vivo experiments, we induced retinal ischemia by unilateral elevation of intraocular pressure for 1 h by direct corneal canulation. Mice were injected with liposomes or PBS 24 h before IR, at the time of surgery, and every 24 h until sacrifice. Transmission electron microscopic analysis was used to identify necrotic and apoptotic cells in ischemic retinas. The changes in expression of pro-inflammatory genes 24 h post reperfusion were assessed by quantitative reverse transcription polymerase chain reaction (RT–PCR). Corresponding changes in protein abundances were analyzed by immunohistochemistry. Cell death was evaluated by direct counting of neurons in the ganglion cell layer (GCL) of flatmounted retinas 7 days post reperfusion.

**Results:**

Treatment with ATP-liposomes increases retinal ganglion cell (RGC) survival and decreases necrotic cell death following OGD. Injection of ATP-liposomes markedly decreased necrotic cell death in the GCL following retinal ischemia. The ATP-liposome treatment reduced the expression of pro-inflammatory genes, including that of interleukin 1β (*Il1β*), interleukin 6 (*Il6*), tumor necrosis factor (*Thf*), chemokine (C-C motif) ligand 2 (*Ccl2*), chemokine (C-C motif) ligand 5 (*Ccl5*), chemokine (C-X-C motif) ligand 10 (*Cxcl10*), intercellular adhesion molecule 1 (*Icam1*), and nitric oxide synthase 2 (*Nos2*), in the retina 24 h after IR and significantly reduced the GCL neuron death rate 7 days after reperfusion.

**Conclusions:**

ATP-liposome treatment of IR-challenged neural tissues suppressed necrosis and correlated with a significantly reduced level of inflammation and retinal damage.

## Introduction

Retinal ischemia results in a prolonged period of cell death with a high level of necrosis versus apoptosis at an early stage of pathology [1-4]. Rather than mere waste disposal, the clearance of cells that are dying by necrosis facilitates distinct signaling in the affected tissue. Necrosis of tissue leads to inflammatory and toxic activation of phagocytes [5-7]. Thus, the predominance of necrotic cell death could mediate additional damage after ischemia-reperfusion (IR) injury. At the same time, reduced necrosis might be a way to improve outcome after IR injury.

The magnitude and form assumed by cell death after ischemia are largely dependent on intracellular levels of ATP (ATP). Apoptosis is ATP dependent in general, and cell death fate by apoptosis or necrosis is determined by intracellular ATP levels [8-10]. In areas where blood flow is limited, there is rapid exhaustion of intracellular ATP due to insufficient oxygen and rapid consumption of glucose, inhibiting apoptosis and inducing necrotic cell death [10,11]. Thus, the application of exogenous ATP could restore the viability of ischemic cells. However, the pharmacological use of ATP is restricted due to poor cellular penetration and rapid hydrolysis by ectoenzymes [12].

In an attempt to develop a system of protecting ATP against degradation during delivery, we considered an encapsulation into multilamellar vesicles (liposomes). The efficiency of the liposomes encapsulated with ATP (ATP-liposomes) in preventing cell death and improving the energy status of cells has been shown in vitro and in vivo [12]. Thus, the recovery of ATP stores delivered by ATP-liposomes would increase the survival of some cells and let others die by apoptosis instead of necrosis. Because necrosis, unlike apoptosis, is associated with a release of intracellular contents and a subsequent inflammatory reaction, reduction in the proportion of necrotic death should result in a smaller final lesion. In this study we investigated the effect of ATP-liposome injection on the survival of retinal neurons in the ganglion cell layer (GCL) challenged with IR injury.

## Methods

### Animals

All experiments and post-surgical care were performed in compliance with the National Institutes of Health Guide for the Care and Use of Laboratory Animals, the Association for Research in Vision and Ophthalmology statement for use of animals in ophthalmic and vision research, and according to the University of Miami Institutional Animal Care and Use Committee approved protocols. All animals used in our experiments were 3-month-old C57BL/6J (stock number 000664; Jackson Laboratory, Bar Harbor, ME) male mice or 10–14-day-old pups.

### Isolation of retinal ganglion cells

Pups were euthanized by cervical dislocation, eyes were enucleated, and retinas were mechanically dissected out. Retinal ganglion cells (RGCs) were isolated according to the two-step immunopanning method [13]. Briefly, the whole retinas were incubated in papain solution (16.5 U/ml) for 30 min. In the next step macrophage and endothelial cells were removed from the cell suspension by panning with the anti-macrophage antiserum (Accurate Chemical, Westbury, NY). RGCs were specifically bound to the panning plates containing anti-Thy1.2 antibody and released by trypsin incubation. RGCs were grown in Neurobasal/B27 media (Invitrogen, Carlsbad, CA).

### Oxygen and glucose deprivation model

RGCs treated with ATP-liposomes, blank liposomes (PC-liposomes), and phosphate buffer solution (PBS: 1.4 mM KH_2_PO_4_, 8 mM Na_2_HPO_4_, 140 mM NaCl, 2.7 mM KCl, pH 7.4; vehicle) were deprived of oxygen using an anaerobic chamber (0% O_2_, 5% CO_2_, and 95% N_2_) and glucose- and sodium pyruvate-free Neurobasal media (Invitrogen) media for 6 h at 37 °C. After oxygen and glucose deprivation, the culture medium was exchanged for fresh Neurobasal/B27 media, and the neurons were further incubated for 12 h in a 5% CO_2_ atmosphere. Parallel cultures were exposed to oxygenated media in a normoxic incubator (37 °C; atmosphere 5% CO_2_) to serve as sham controls.

### Transient retinal ischemia

Retinal ischemia was induced for 60 min by introducing into the anterior chamber of the eye a 33-gauge needle attached to a normal (0.9% NaCl) saline-filled reservoir raised above the animal to increase intraocular pressure (IOP; IOP increased to 120 mmHg). The contralateral eye was canulated and maintained at normal IOP to serve as a normotensive control. Body temperature was maintained at 37±0.5 °C. Complete retinal ischemia, evidenced by a whitening of the anterior segment of the eye and blanching of the retinal arteries, was verified by microscopic examination.

### Treatment with liposomes

Liposomes encapsulated with carboxyfluorescein (CF-liposomes), ATP-liposomes, and PC-liposomes with a diameter of about 100 nm were prepared by Encapsula NanoSciences (Nashville, TN). In brief, a mixture of 12 mM L-[α]-phosphatidylserine (PS) and 33 mM L-[α]-phosphatidylcholine (PC) in chloroform were placed in a test tube. The liposomes were composed of either PC only (PC-liposomes) or a combination of PC and PS at a molar ratio of 7:3 (PS-liposomes). The solvent was removed in a rotary evaporator at 30 °C under reduced pressure and then dried by a desiccator for 1 h. The desiccated lipids were dispersed with a vortex mixer in PBS (pH 7.4) to obtain a final concentration of 10 mg/ml total lipids. The lipid suspensions were subsequently sonicated for 10 min on ice. The liposome solutions were centrifuged, and then the supernatants were used for the experiments. Mice were injected intramuscularly (IM) with either liposomes (0.5 mg [or 13.2 mM] suspension in PBS per animal) or carrier buffer (PBS) 24 h before IR, at the time of surgery, and then every 24 h until sacrifice. Retinas were collected and analyzed 1 or 7 days after IR. We used six animals in each treated group. For in vitro experiments RGCs were treated with 200 µM liposomes during oxygen and glucose deprivation (OGD) and reperfusion at 37 °C.

### Neuronal death assay

After OGD, necrotic and apoptotic cells were determined using the Vybrant Apoptosis Assay Kit #2 (Invitrogen, Carlsbad, CA). Cells were imaged using a Leica TSL AOBS SP5 confocal microscope (Leica Microsystems, Exton, PA) and counted using MetaMorph (Molecular Devices, Sunnyvale, CA) software. The percentage of necrotic cells (annexin V and propidium iodide [PI]) and apoptotic cells (only annexin V) relative to the total number of cells was determined for each of ten images.

### ATP level in ischemic retina

Animals were perfused with PBS. Retinas were removed and transferred to a 1.5-ml microfuge tube, and 10 µl of ice-cold 0.4 M perchloric acid was added per milligram wet tissue. The retinas were immediately homogenized with a pellet pestle. The acidic homogenate was kept on ice for 30 min and then centrifuged at 16,100× g at 4 °C for 10 min. The supernatant was neutralized with 10 µl of 4 M K_2_CO_3_ added to 100 µl of the supernatant, kept on ice for 10 min and at −8 °C for 1–2 h to promote precipitation of the perchlorate, and then centrifuged again. Supernatants were stored at −8 °C until the luciferase assay. The ATP concentration was assessed quantitatively by using the EnzyLight™ ATP Assay Kit (BioAssay Systems, Hayward, CA) according to the instructions. Briefly, 100 μl of a fivefold dilution of each sample was transferred in triplicate into wells of a white opaque 96-well plate (Costar, Corning, NY) and incubated for 10 min with 90 μl of reconstituted reagent containing the assay buffer, d-luciferin, and luciferase (95:1:1). Luminescence was read on a Centro XS3 LB960 luminometer (Berthold Technologies, Oak Ridge, TN) with an integration time of 1 s per well. ATP concentrations were extrapolated from the linear ATP standard curve.

### Transmission electron microscopy

Retinas with ischemia reperfusion were fixed with 2.5% glutaraldehyde in 0.1 M phosphate buffer (pH 7.4) for 1 h at 25 °C, postfixed with 1% osmium tetroxide for 1 h at 25 °C, dehydrated through a graded alcohol series, and embedded in Epon812 resin. Ultrathin sections (80 nm thick) were cut with an Ultracut S (Leica, Vienna, Austria) and then stained with uranyl acetate and lead citrate for 30 and 5 min, respectively. The stained sections were observed under a Philips CM-10 transmission electron microscope (FEI Company, Hillsboro, Oregon). Transmission electron microscopic (TEM) analysis was used to characterize the cell death mode in the retinas at 24 h after the ischemia reperfusion. The numbers of necrotic and apoptotic cells for these measurements, taken in 10 adjacent areas (one area, 8×12 μm) within 3 mm of the optic nerve, were calculated.

### Real-time PCR

Real-time PCR analysis was performed using gene-specific primers (Table 1). Total RNA was extracted from retinas using Nanoprep (Stratagene, Carlsbad, CA) and reverse transcribed with Superscript III (Invitrogen) polymerase to synthesize cDNA. Real-time PCR was performed in the Rotor-Gene 6000 Cycler (Qiagen, Valencia, CA) using the SYBR GREEN PCR MasterMix (Qiagen). For each gene, relative expression was calculated by comparison with a standard curve following normalization to the housekeeping gene β-actin (*Actb*) expression chosen as control.

**Table 1:** List of PCR primers and the primary antibodies

**Gene**	**Oligonucleotides**	**PCR product size**	**Primary antibodies**
*Il1b*	F: GACCTTCCAGGATGAGGACA	283 bp	PR-427β (Endogen)
	R: AGGCCACAGGTATTTTGTCG		
*Il6*	F: ATGGATGCTACCAAACTGGAT	138 bp	AMC0864 (Biosource)
	R: TGAAGGACTCTGGCTTTGTCT		
*Il10*	F: GGTTGCCAAGCCTTATCGGA	190 bp	
	ACCTGCTCCACTGCCTTGCT		
*Tgfb1*	F: TGAGTGGCTGTCTTTTGACG	292 bp	
	R: TCTCTGTGGAGCTGAAGCAA		
*Ccl2*	F: AGGTCCCTGTCATGCTTCTG	279 bp	Sc-1784 (Santa Cruz)
	R: ATTTGGTTCCGATCCAGGTT		
*Ccl5*	F: AGCAGCAAGTGCTCCAATCT	280 bp	
	R: ATTTCTTGGGTTTGCTGTGC		
*Cxcl10*	F: GCTGCAACTGCATCCATATC	293 bp	Sc-1406 (Santa Cruz)
	R: CACTGGGTAAAGGGGAGTGA		
*Icam1*	F: TGGTGATGCTCAGGTATCCA	273 bp	
	R: CACACTCTCCGGAAACGAAT		
*Vcam1*	F: GTGGTGCTGTGACAATGACC	287 bp	
	R: ACGTCAGAACAACCGAATCC		
*Cybb*	F: GACTGCGGAGAGTTTGGAAG	277 bp	
	R: ACTGTCCCACCTCCATCTTG		
*Nos2*	F: CAGAGGACCCAGAGACAAGC	299 bp	
	R: TGCTGAAACATTTCCTGTGC		
*Actb*	F: CACCCTGTGCTGCTCACC	327 bp	
	R: GCACGATTTCCCTCTCAG		

### Immunohistochemistry

Fixed retinas were sectioned to a thickness of 100 μm with vibratome (Vibratome, St. Louis, MO) and immunostained using the protocol described earlier [13]. Briefly, sections were permeabilized with 0.3% Triton X-100 in PBS for 45 min, rinsed in PBS and blocked by 5% donkey serum, 2% BSA and 0.15% Tween-20 in PBS for 1 h and incubated overnight with various primary antibodies (Table 1), followed by species-specific secondary fluorescent antibodies (AlexaFluor; Invitrogen). Control sections were incubated without primary antibodies. Imaging was performed with a Leica TSL AOBS SP5 confocal microscope (Leica Microsystems, Exton, PA).

### Immunohistochemistry for Neuronal Nuclei (NeuN) in flatmounted retinas

Eyes were enucleated upon euthanasia by CO_2_ inhalation under anesthesia, incised at the ora serrata, immersion fixed in a 4% paraformaldehyde solution (in PBS, pH 7.4) for 1 h, and the retinas removed. The retinas were cryoprotected overnight in 30% sucrose followed by 3 freeze–thaw cycles, rinsed 3×10 min in 0.1 M PBS and blocked by 5% donkey serum, 0.1% Triton X-100 in 0.1 M Tris buffer (TB) for 1 h, and incubated overnight with monoclonal fluorescein isothiocyanate (FITC)-conjugated NeuN antibody (dilution 1:300; Chemicon, Billerica, MA). After 3×10 min rinses in 0.1 M TB, retinas were flatmounted, coverslipped, and imaged using a Leica TSL AOBS SP5 confocal microscope.

### Counting of NeuN positive ganglion cell layer (GCL) neurons

NeuN-positive neurons in the GCL, including RGCs and displaced amacrine cells, were imaged by confocal microscopy in flatmounted retinas. Individual retinas were sampled randomly to collect a total of 20 images located at the same eccentricity in the four retinal quadrants using a 20× objective lens. NeuN-positive neurons were counted semi-automatically using MetaMorph (Molecular Devices, Sunnyvale, CA) software. Cell loss in the ischemic retinas was calculated as percentile of the mean cell density in fellow control eyes.

### Statistical analysis

Statistical analysis of real-time PCR and cell density data was performed with one-way ANOVA followed by the Tukey test for multiple comparisons. In case of single comparisons, the Student *t* test was applied. P values equal to or less than 0.05 were considered statistically significant.

## Results

### Treatment with ATP-liposomes increases RGC survival and decreases necrotic cell death following OGD

**Figure 1:**
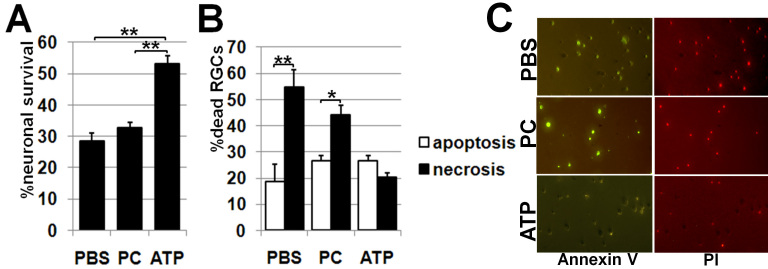
ATP-liposomes rescue retinal ganglion cell from necrosis after oxygen and glucose deprivation. **A**: Treatment by ATP (ATP)-liposomes results in neuroprotective effects in the retinal ganglion cell (RGC) primary cultures after 6 h of oxygen and glucose deprivation (OGD) following 12 h re-oxygenation compared to phosphatidylcholine (PC)-liposomes and PBS-treated RGCs; survival rate after OGD is expressed as a percentage of the mean value obtained in parallel cultures. **B**: Treatment with ATP-liposomes decreases necrotic cell death in the RGCs cultures following 6 h OGD and 12 h re-oxygenation. C: Necrotic and apoptotic cells were determined using Annexin V as a marker of apoptotic cells and Annexin V/propidium iodide (PI) to identify necrotic cells. The percentage of necrotic cells and apoptotic cells relative to the total number of cells was determined for each of ten images (*p<0.05, **p<0.01).

OGD, a model of ischemia in vitro, produces a rapid decrease of neuronal ATP followed by cell death by necrosis and apoptosis [12,14]. To restore the required level of ATP in ischemic cells, ATP-liposomes were applied. We induced OGD in cultures of primary RGCs, which were purified using the two-step immunopanning protocol. Because ATP is unstable, we used ATP-liposomes prepared within 24 h for each experiment. Cultures of primary RGCs were assessed for levels of necrotic and apoptotic cells and survival after 12 h using annexin V as a marker of apoptotic cells and annexin V/PI to identify necrotic cells (Figure 1C). Quantification of cell death was performed by phase-contrast microscopy and showed significantly higher survival in OGD-exposed cultures treated by ATP-liposomes versus PC-liposomes or PBS (p<0.01, Figure 1A). The percentage of necrotic cells was significantly higher in cultures treated with PC-liposomes or PBS versus ATP-liposomes (p<0.01, Figure 1B). Thus, treatment with ATP-liposomes reduced OGD cell death by necrosis and increased the level of cell survival.

### Treatment with ATP-liposomes reduces inflammation following retinal ischemia

**Figure 2:**
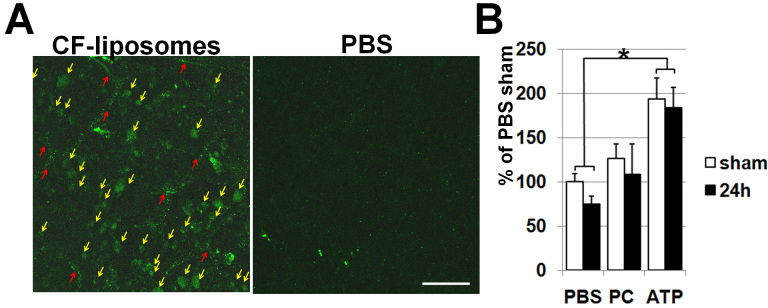
ATP-liposomes effectively pass through the blood–retinal barrier. **A**: Confocal photomicrographs of flatmounted ischemic retinas treated with carboxyfluorescein (CF)-liposomes or carrier buffer PBS were taken 24 h after ischemia reperfusion (IR). Nonfixed freshly isolated retinas were visualized by confocal microscopy in the ganglion cell layer (GCL). Retinal GCL cells display green fluorescence, indicating liposomal uptake (yellow arrows). Punctuate traces of CF labeling are likely endothelial cells in blood capillaries (red arrows). PBS-treated controls display little autofluorescence. The scale bar represents 50 µm. **B**: Relative changes in ATP (ATP) content in control (sham) and ischemic (24 h) retinas following the treatment with ATP, PC- liposomes, and PBS 24 h after reperfusion. The results are shown as a percentage of the corresponding value in PBS-treated sham-operated retinas (*p<0.05, n=5).

It has been shown that liposomes are efficiently incorporated into the central nervous system across the blood–brain barrier of normal and post-ischemic tissues [15]. To investigate liposomal incorporation into the ischemic retina across the blood–retinal barrier, animals were IM injected with liposomes encapsulated with carboxyfluorescein (CF-liposomes) 24 h before IR and at the time of surgery. Retinal IR was induced by unilateral elevation of IOP via corneal canulation with normotensive saline. Retinas were collected and analyzed 24 h after IR. After IM administration of CF-liposomes, numerous fluorescent cells were seen in the ischemic retinas (Figure 2A). In addition, the level of ATP in ischemic retinas treated with ATP, PC-liposomes, and PBS was assessed using the ATP bioluminescent assay. We injected experimental mice IM with ATP-liposomes twice: 24 h before IR and at the time of surgery. Because ATP is unstable we used ATP-liposomes prepared within 24 h for each experiment. Control animals were injected with PC-liposomes at equimolar concentration or PBS. The contralateral eye served as a normotensive control. We did not detect statistically significant ATP depletion in treatment with ATP-liposomes, PC-liposomes, and PBS ischemic retinas compared to sham-operated retinas 24 h after reperfusion (Figure 2B). However, liposomal ATP significantly increased the level of ATP in ischemic and sham-operated retinas compared to PC-liposomes and PBS treatment (Figure 2B). Thus, ATP-liposomes used in our study effectively pass through the blood–retinal barrier.

**Figure 3:**
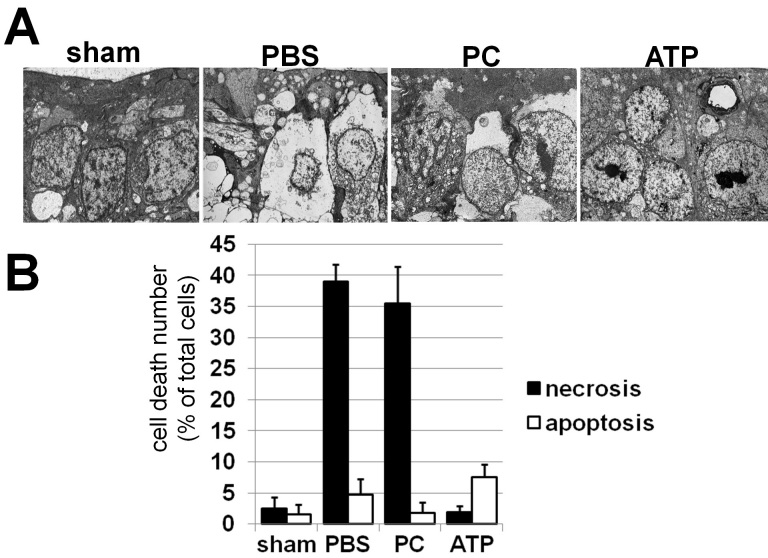
ATP-liposomes prevent retinal ischemia-induced necrosis. **A**: Transmission electron microscopic (TEM) analysis of the ganglion cell layer (GCL) of sham operated (sham) and ischemic retinas treated with ATP (ATP)-, phosphatidylcholine (PC)-liposomes, and PBS 24 h after reperfusion was used to identify necrotic and apoptotic cells. **B**: Quantitative comparisons in terms of the cell populations showed characteristic features of necrotic (closed column) and apoptotic (open column) cells detected by the analysis of the TEM micrographs. Results represent the mean±standard error of the mean of three to five independent experiments (*p<0.05).

Our in vitro experiments suggest that treatment of ischemic retinas with ATP-liposomes could reduce necrotic cell death. To test this hypothesis, we used TEM analysis, which has been considered a “gold standard” in cell-death research. Retinal ischemia was induced, and animals were treated as above. Twenty-four hours after reperfusion, retinas were collected and used for TEM analysis. Our results suggest that GCL cells in ischemic retinas treated with PBS and PC-liposomes died predominantly by necrosis, which was characterized by a loss of electron density in the cytosol without nuclear condensation (Figure 3). At the same time, injection of ATP-liposomes markedly reduced necrotic cell death in the GCL following retinal ischemia (Figure 3). It should be noted that treatment with ATP-liposomes significantly decreased edema in retinas after IR injury (data not shown).

**Figure 4:**
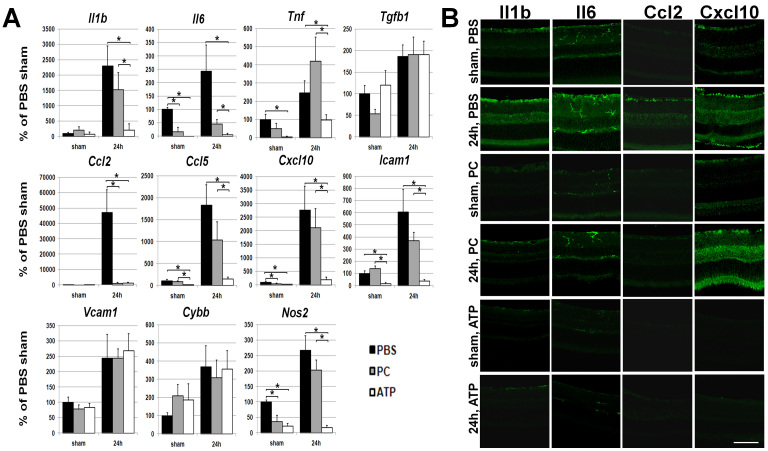
ATP-liposome treatment reduces inflammatory gene and protein expression following ischemia-reperfusion (IR) injury **A**: Differential expression of cytokines, chemokines, cell adhesion molecules, and nitric oxide (NO) synthase was assessed in sham-operated (sham) controls and in the ischemic retinas of ATP (ATP)-, phosphatidylcholine (PC)-liposome-, and PBS-treated animals 24 h after reperfusion. Gene expression was assessed using real-time PCR in sham-operated controls and experimental retinas following IR. For each gene, results are expressed as a percentage of corresponding value in the sham-operated eye of PBS-treated animals±standard error of the mean after normalization to β-actin (*p<0.05, six animals per group). **B**: Immunohistochemistry for the Il1b, Il6, Ccl2, and Cxcl10 protein accumulation in post-ischemic retinas of ATP-, PC-liposome-, and PBS-treated mice is consistent with increased levels of the transcripts at the level of corresponding proteins. Scale bar indicates 100 μm.

Necrotic cell death, in contrast to apoptotic cell death, leads to the release of “danger” signals initiating an innate immune response. Thus, in tissue treated by ATP-liposomes, reduced necrotic cell death could contribute to a diminished inflammatory response in tissue after IR injury. To test this hypothesis, we analyzed total RNA extracted from the IR-exposed retinas for the abundance of transcript for pro-inflammatory genes. Retinas were analyzed 24 h post reperfusion because most changes in gene expression for pro-inflammatory factors typically occur shortly after IR injury. Transcriptional upregulation of cytokines (interleukin 1β [*Il1β*], interleukin 6 [*Il6*], and tumor necrosis factor [*Thf*]), chemokines (chemokine (C-C motif) ligand 2 [*Ccl2*], chemokine (C-C motif) ligand 5 [*Ccl5*], and chemokine (C-X-C motif) ligand 10 [*Cxcl10*]), intercellular adhesion molecule 1 (*Icam1*), and nitric oxide synthase 2 (*Nos2*) was evident in experimental eyes of animals treated with either ATP-, PC-liposome, or PBS (Figure 4A). However, the level of activation for these genes, particularly for *Il1β, Il6*, *Tnf, Ccl5, Cxcl10, Icam1,* and *Nos2* genes, was significantly lower in the mice treated with ATP-liposomes versus PC-liposomes or PBS. As for transforming growth factor beta 1 (*Tgfb1*), *Ccl2*, vascular cell adhesion molecule 1 (*Vcam1*), and cytochrome b-245, beta polypeptide (*Cybb*) genes, the difference between ATP- and PC-liposome treatments was not statistically significant. These data were confirmed for *Ccl2* and *Cxcl10* at the protein accumulation level, as detected in ischemic retina 24 h after reperfusion by immunohistochemistry (Figure 4B). These results indicate that ATP liposomes are more potent in suppressing inflammatory pathways compared to PC liposomes. Nevertheless, significant suppression of inflammatory genes was also evident with PC-liposome treatment, the effect that we and others described earlier [16].

### Treatment with ATP-liposomes reduces neuronal loss in retinal ischemia

**Figure 5:**
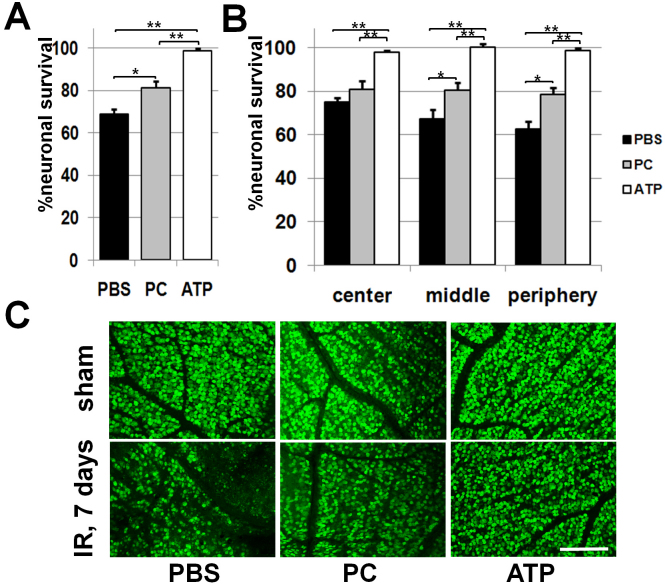
Treatment by ATP-liposomes results in neuroprotective effects in the ganglion cell layer of retinas after ischemia reperfusion. **A**: The percentage survival of ganglion cell layer (GCL) neurons in the ischemic retinas of ATP (ATP)-, phosphatidylcholine (PC)-liposome-, and PBS-treated animals were detected 7 days after reperfusion (*p<0.05, **p<0.01, n=6). **B**: The percentage of Neuronal Nuclei (NeuN)-labeled neurons in regions of central, middle, and peripheral retina were compared between sham-operated (sham) and ischemic eyes of ATP-, PC liposome-, and PBS-treated animals 7 days after reperfusion (*p<0.05, **p<0.01, n=6). **C**: Representative confocal images of NeuN-labeled GCLs (green) in flatmounted controls (sham) and ischemic retinas were taken 7 days after reperfusion. Scale bar indicates 100 μm.

IR-induced degeneration of neurons in the GCL is biphasic with a primary degeneration occurring within 24 h after reperfusion and a secondary degeneration progressing over several days [17]. To detect cumulative damage from both waves of degeneration, we evaluated neuronal survival 1 week after reperfusion. Mice were injected IM with liposomes or PBS 24 h before IR, at the time of surgery, and then every 24 h. We evaluated neuronal cell death by measuring the density of neurons labeled with the neuronal marker NeuN in the GCL in flatmounted retinas. The percentage of surviving GCL neurons in the IR retinas was significantly higher in mice injected with ATP-liposomes (99±1%) compared to those injected with PC-liposomes (81±3%, p<0.01, n=6) and PBS (69±2%, p<0.001, n=6; Figure 5A). The NeuN immunohistochemistry showed that affected neurons were distributed evenly, without a geographic pattern, across ischemic retinas in all treatment groups (Figure 5B,C).

## Discussion

In this work we used liposomes as a vehicle for ATP delivery to retinal cells. We applied ATP-liposomes in vitro and in vivo to test whether restoration of intracellular ATP levels protects retinal neurons against IR injury. Our results showed that treatment with ATP-liposomes resulted in significant increase in survival of neurons challenged with IR. Such increased tolerance of inner retinal neurons to IR injury correlated with increased levels of ATP in the retinas of treated animals and reduced cell death by necrosis in both cultured cells and in ischemic retinas. Thus, our results support the hypothesis that sufficient levels of intracellular ATP delivered by liposomes protect cells from energy failure caused by IR injury. This is in a good agreement with studies where ATP-liposomes greatly increased the number of ischemic episodes tolerated before brain electrical silence and death appeared [18]. Significantly, it was shown that ATP-liposomes can effectively protect myocardium from IR damage [19,20].

Inflammation is a pathologic hallmark of IR injury that spatiotemporally correlates to the delayed phase of cell death [19-22]. At the cellular level, the postischemic inflammatory response is facilitated by phagocytic cells of the innate nonspecific immune system [19-22]. The predominance of necrotic cells and abundance of necrotic factors mediate inflammatory stress, which can be responsible for additional damage after IR injury. Our results suggest that near-complete blockade of necrosis in the GCL of animals treated with ATP-liposomes correlated with a significant decrease in pro-inflammatory gene expression. These included pro-inflammatory cytokines, chemokines, cell adhesion molecules, and NO synthase. A robust inflammatory response in PBS- and PC-liposome-treated control animals can exacerbate the injury-induced stress by over exposing neurons to neurotoxic levels of Tnf, Il1b, and Il6 cytokines, as shown previously [23]. The Ccl5 and Cxcl10 chemokines and the cell adhesion molecules Icam1 are essential for immune cell activation, attraction, and trafficking across the blood–brain barrier into the central nervous system under both physiologic and pathological conditions [24,25]. Decreased activity of genes encoding these molecules in animals treated with ATP-liposomes suggests that the diminished ability for inflammatory cells to infiltrate the retina could elicit a neuroprotective effect following ischemia. Finally, an excessive activity of *Nos2*, encoding inducible NO-synthase, is likely causing oxidative stress. The activation of this enzyme is broadly deleterious, and its inhibition was shown to be neuroprotective [26]. Our analysis of IR-challenged retinas revealed that gene expression of *Nos2* was suppressed in the retina of ATP-liposome-treated animals relative to controls. Thus, reduced level of inflammation in ischemic retinas treated with ATP-liposomes could affect increased cumulative survival of GCL neurons. We hypothesize that in combination with developed antibody targeting to retinal neurons, ATP-immunoliposome treatment may represent a perspective new strategy for retinal ischemia.

In conclusion, this work represents the first report on protection from IR injury in the retina induced by ATP-liposomes. Our results suggest that suppressing neuronal necrosis using ATP-liposomes in the IR-challenged neural tissues could promote a neuroprotective environment and reduce tissue damage.

## References

[1] Fujita R Ueda M Fujiwara K Ueda H Prothymosin-alpha plays a defensive role in retinal ischemia through necrosis and apoptosis inhibition. Cell Death Differ 2009 16 349 58 18989338 10.1038/cdd.2008.159

[2] Osborne NN Casson RJ Wood JP Chidlow G Graham M Melena J Retinal ischemia: mechanisms of damage and potential therapeutic strategies. Prog Retin Eye Res 2004 23 91 147 14766318 10.1016/j.preteyeres.2003.12.001

[3] Lafuente MP Villegas-Perez MP Selles-Navarro I Mayor-Torroglosa S Miralles de Imperial J Vidal-Sanz M Retinal ganglion cell death after acute retinal ischemia is an ongoing process whose severity and duration depends on the duration of the insult. Neuroscience 2002 109 157 68 11784707 10.1016/s0306-4522(01)00458-4

[4] Osborne NN Ugarte M Chao M Chidlow G Bae JH Wood JP Nash MS Neuroprotection in relation to retinal ischemia and relevance to glaucoma. Surv Ophthalmol 1999 43 Suppl 1 S102 28 10416754 10.1016/s0039-6257(99)00044-2

[5] Vanlangenakker N Berghe TV Krysko DV Festjens N Vandenabeele P Molecular mechanisms and pathophysiology of necrotic cell death. Curr Mol Med 2008 8 207 20 18473820 10.2174/156652408784221306

[6] Fadok VA Bratton DL Guthrie L Henson PM Differential effects of apoptotic versus lysed cells on macrophage production of cytokines: role of proteases. J Immunol 2001 166 6847 54 11359844 10.4049/jimmunol.166.11.6847

[7] Krysko DV D'Herde K Vandenabeele P Clearance of apoptotic and necrotic cells and its immunological consequences. Apoptosis 2006 11 1709 26 16951923 10.1007/s10495-006-9527-8

[8] Miyoshi N Oubrahim H Chock PB Stadtman ER Age-dependent cell death and the role of ATP in hydrogen peroxide-induced apoptosis and necrosis. Proc Natl Acad Sci USA 2006 103 1727 31 16443681 10.1073/pnas.0510346103 PMC1413652

[9] Leist M Single B Castoldi AF Kuhnle S Nicotera P Intracellular adenosine triphosphate (ATP) concentration: a switch in the decision between apoptosis and necrosis. J Exp Med 1997 185 1481 6 9126928 10.1084/jem.185.8.1481 PMC2196283

[10] Eguchi Y Shimizu S Tsujimoto Y Intracellular ATP levels determine cell death fate by apoptosis or necrosis. Cancer Res 1997 57 1835 40 9157970

[11] Taoufik E Probert L Ischemic neuronal damage. Curr Pharm Des 2008 14 3565 73 19075733 10.2174/138161208786848748

[12] Puisieux F Fattal E Lahiani M Auger J Jouannet P Couvreur P Delattre J Liposomes, an interesting tool to deliver a bioenergetic substrate (ATP). in vitro and in vivo studies. J Drug Target 1994 2 443 8 7704489 10.3109/10611869408996820

[13] Ivanov D Dvoriantchikova G Nathanson L McKinnon SJ Shestopalov VI Microarray analysis of gene expression in adult retinal ganglion cells. FEBS Lett 2006 580 331 5 16376886 10.1016/j.febslet.2005.12.017

[14] Plesnila N Zinkel S Le DA Amin-Hanjani S Wu Y Qiu J Chiarugi A Thomas SS Kohane DS Korsmeyer SJ Moskowitz MA BID mediates neuronal cell death after oxygen/ glucose deprivation and focal cerebral ischemia. Proc Natl Acad Sci USA 2001 98 15318 23 11742085 10.1073/pnas.261323298 PMC65027

[15] Chapat S Frey V Claperon N Bouchaud C Puisieux F Couvreur P Rossignol P Delattre J Efficiency of liposomal ATP in cerebral ischemia: bioavailability features. Brain Res Bull 1991 26 339 42 2049600 10.1016/0361-9230(91)90004-4

[16] Hashioka S Han YH Fujii S Kato T Monji A Utsumi H Sawada M Nakanishi H Kanba S Phosphatidylserine and phosphatidylcholine-containing liposomes inhibit amyloid beta and interferon-gamma-induced microglial activation. Free Radic Biol Med 2007 42 945 54 17349923 10.1016/j.freeradbiomed.2006.12.003

[17] Sellés-Navarro I Villegas-Perez MP Salvador-Silva M Ruiz-Gomez JM Vidal-Sanz M Retinal ganglion cell death after different transient periods of pressure-induced ischemia and survival intervals. A quantitative in vivo study. Invest Ophthalmol Vis Sci 1996 37 2002 14 8814140

[18] Laham A Claperon N Durussel JJ Fattal E Delattre J Puisieux F Couvreur P Rossignol P Intracarotidal administration of liposomally-entrapped ATP: improved efficiency against experimental brain ischemia. Pharmacol Res Commun 1988 20 699 705 3212008 10.1016/s0031-6989(88)80117-6

[19] Verma DD Hartner WC Levchenko TS Bernstein EA Torchilin VP ATP-loaded liposomes effectively protect the myocardium in rabbits with an acute experimental myocardial infarction. Pharm Res 2005 22 2115 20 16258743 10.1007/s11095-005-8354-x

[20] Verma DD Levchenko TS Bernstein EA Torchilin VP ATP-loaded liposomes effectively protect mechanical functions of the myocardium from global ischemia in an isolated rat heart model. J Control Release 2005 108 460 71 16233928 10.1016/j.jconrel.2005.08.029 PMC1634739

[21] Lipton P Ischemic cell death in brain neurons. Physiol Rev 1999 79 1431 568 10508238 10.1152/physrev.1999.79.4.1431

[22] Dvoriantchikova G Barakat D Brambilla R Agudelo C Hernandez E Bethea JR Shestopalov VI Ivanov D Inactivation of astroglial NF-kappaB promotes survival of retinal neurons following ischemic injury. Eur J Neurosci 2009 30 175 85 19614983 10.1111/j.1460-9568.2009.06814.x PMC2778328

[23] Raivich G Bohatschek M Kloss CU Werner A Jones LL Kreutzberg GW Neuroglial activation repertoire in the injured brain: graded response, molecular mechanisms and cues to physiological function. Brain Res Brain Res Rev 1999 30 77 105 10407127 10.1016/s0165-0173(99)00007-7

[24] Ubogu EE Cossoy MB Ransohoff RM The expression and function of chemokines involved in CNS inflammation. Trends Pharmacol Sci 2006 27 48 55 16310865 10.1016/j.tips.2005.11.002

[25] Connolly ES Jr Winfree CJ Springer TA Naka Y Liao H Yan SD Stern DM Solomon RA Gutierrez-Ramos JC Pinsky DJ Cerebral protection in homozygous null ICAM-1 mice after middle cerebral artery occlusion. Role of neutrophil adhesion in the pathogenesis of stroke. J Clin Invest 1996 97 209 16 8550836 10.1172/JCI118392 PMC507081

[26] Neufeld AH Kawai S Das S Vora S Gachie E Connor JR Manning PT Loss of retinal ganglion cells following retinal ischemia: the role of inducible nitric oxide synthase. Exp Eye Res 2002 75 521 8 12457864 10.1006/exer.2002.2042

